# From Rigid Order to Radical Variation: Mitogenome Evolution in the Main Lineages of a Lesser-Known Animal Phylum (Gastrotricha)

**DOI:** 10.1093/gbe/evag001

**Published:** 2026-01-10

**Authors:** Anush Kosakyan, Leandro Gammuto, Agata Cesaretti, Francesco Saponi, Valentina Serra, Giulio Petroni, Jan-Niklas Macher, Oscar Wallnoefer, Federico Plazzi, M Antonio Todaro

**Affiliations:** Department of Life Sciences, University of Modena and Reggio Emilia, Modena, Italy; National Biodiversity Future Center (NBFC), Palermo, Italy; Department of Biology and Biotechnology “Lazzaro Spallazani,” University of Pavia, Pavia, Italy; Department of Life Sciences, University of Modena and Reggio Emilia, Modena, Italy; Department of Life Sciences, University of Modena and Reggio Emilia, Modena, Italy; National Biodiversity Future Center (NBFC), Palermo, Italy; Department of Earth and Marine Sciences, University of Palermo, Palermo, Italy; Dipartimento of Biology, University of Pisa, Pisa, Italy; Dipartimento of Biology, University of Pisa, Pisa, Italy; Interdepartmental Center for Electron Microscopy (CIME), University of Pisa, Pisa, Italy; Center for Instrument Sharing of the University of Pisa (CISUP), University of Pisa, Pisa, Italy; Naturalis Biodiversity Center, Leiden, The Netherlands; Department of Environmental Biology, Institute of Environmental Sciences (CML), Leiden University, Leiden, The Netherlands; Department of Biological, Geological and Environmental Sciences, University of Bologna, Bologna, Italy; Department of Biological, Geological and Environmental Sciences, University of Bologna, Bologna, Italy; Department of Life Sciences, University of Modena and Reggio Emilia, Modena, Italy; National Biodiversity Future Center (NBFC), Palermo, Italy

**Keywords:** evolutionary adaptations, Gastrotricha, mitochondrial genomes, mtDNA modifications, mitochondrial phylogenetics

## Abstract

Mitochondrial genomes offer valuable insights into biological and phylogenetic processes, yet the factors shaping their architecture across metazoan lineages remain poorly understood, largely due to limited taxonomic sampling. To address this gap, we analyzed mitochondrial genomes from 20 species spanning a broad taxonomic spectrum of the phylum Gastrotricha. Our findings, supported by phylogenetic analyses based on mitochondrial datasets, reveal two distinct evolutionary patterns: one lineage displays remarkable conservation in genome structure, while the other exhibits variability in gene content, arrangement, strand polarity, and repeat abundance. These contrasting patterns appear to be related to differences in reproductive strategies (hermaphroditism vs. parthenogenesis) and ecological habitats (marine vs. freshwater). While these associations are intriguing, further data are needed to understand the underlying processes. This study highlights the importance of broad phylum-scale mitogenomic sampling for uncovering genomic diversity and advancing our understanding of mitochondrial evolution across Metazoa.

Significance Mitochondrial genomes are widely used to study animal evolution, yet their structural diversity remains poorly understood due to limited sampling across many groups. One such group is Gastrotricha, a little-known phylum of aquatic invertebrates, for which mitochondrial data are very limited (available only for 2 species out of 900 known species). This study generated and analyzed 21 mitogenomes, revealing lineage-specific patterns possibly linked to the reproductive mode and habitat of these organisms. While these associations are preliminary and might be driven by phylogenetic nonindependence, they offer intriguing insights into how ecological and life history traits may correlate with genome architecture. These findings underscore the importance of broader taxonomic sampling to uncover the mechanisms driving mitochondrial evolution in overlooked animal lineages.

## Introduction

The metazoan mitochondrial genome was traditionally believed to be a circular chromosome, ∼14 to 20 kb in length, containing 13 protein-coding genes, 2 ribosomal RNA genes, and 22 tRNA genes (Boore 1999; Saccone et al. 1999; Gissi et al. 2008). Over time, as mitochondrial genomes were sequenced for hundreds of metazoans, many species showed deviations from this conserved structure (Lavrov and Pett 2016; Smith 2016; Shtolz and Mishmar 2023; Struck et al. 2023). These deviations include variations in circular versus linear structure, gene order conservation, gene numbers, and, in some cases, the complete absence of mitochondria (Yahalomi et al. 2020). Information gathered from these mitochondrial genomes has significantly contributed to evolutionary and phylogenetic studies (Gibb et al. 2016; Allio et al. 2017; Dowling and Wolff 2023). The higher mutation rate of mitochondrial DNA compared with nuclear DNA, combined with conserved sites within genes like mtCOI that enable universal primer design, has facilitated the development of barcodes that further our understanding of species relationships (Hebert et al. 2003). Additionally, mitochondrial datasets have played an integral role in advancing phylogeographic studies (Morin et al. 2010), paleogenomics (Nesheva 2014; Posth et al. 2023), population genetics (Lake et al. 2024), genome evolution studies (Butenko et al. 2024), and more. However, despite its extensive use, there are still many metazoan lineages with limited or no available mitochondrial genomic data. One such lineage is the phylum Gastrotricha Metschnikoff (1865).

Gastrotrichs are microscopic, free-living aquatic animals that thrive in sediments at the bottom of marine and freshwater environments. Ranging in size from 80 μm to 3.8 mm, they play an important ecological role in aquatic systems as essential components of food webs (Todaro et al. 2019, 2025; Todaro and Luporini 2022; Souid et al. 2025). Currently, over 900 species of gastrotrichs are classified into two orders: Macrodasyida, which includes ∼385 predominantly marine or estuarine species with only four exceptions, and Chaetonotida, which comprises about 520 mainly freshwater species (Gammuto et al. 2024; Saponi et al. 2024, 2026; Saponi and Todaro 2024; Minowa et al. 2025). It has been suggested that about one-fourth of Chaetonotida species have reinvaded marine environments during their evolution (Kolicka et al. 2020).

Gastrotrichs exhibit diverse adaptations to marine and freshwater habitats and possess a fascinating array of reproductive strategies. Generally, chaetonotidan species are mostly found in freshwater and are parthenogenetic, while macrodasyidan species are primarily marine and hermaphroditic, though exceptions exist in both groups (Kieneke and Schmidt-Rhaesa 2015; Cesaretti et al. 2024). For example, within the Macrodasyida order, the freshwater genus *Redudasys* (with three species), the marine genera *Anandrodasys* (one species), and *Urodasys viviparus* reproduce through parthenogenesis. In contrast, in the Chaetonotida order, notable exceptions include species from the families Neodasyidae (4 species), Muselliferidae (8 species), and Xenotrichulidae (26 species), all of which are primarily marine and exhibit hermaphroditism (Todaro et al. 2019).

Despite their intriguing biology, the evolutionary history of gastrotrichs is not fully resolved, as it is primarily inferred from morphological data. Although molecular markers are available for a relatively high proportion of known species (∼21% as of NCBI 20.02.2025), these data are often limited to just a few genes, such as 18S rDNA, 28S rDNA, and mtCOI. Additionally, transcriptomic data are available for only seven species (Struck et al. 2014; Egger et al. 2015; Laumer et al. 2015), and complete mitochondrial genomes are available for only two species (Golombek et al. 2015; Gammuto et al. 2024), which presents a significant challenge to understanding the deep evolutionary history of the group. Recently, the first gastrotrich genome was published, once again confirming the phylogenetic position of Gastrotricha as a sister taxon to Platyhelminthes (Roberts et al. 2024).

**Fig. 1.**
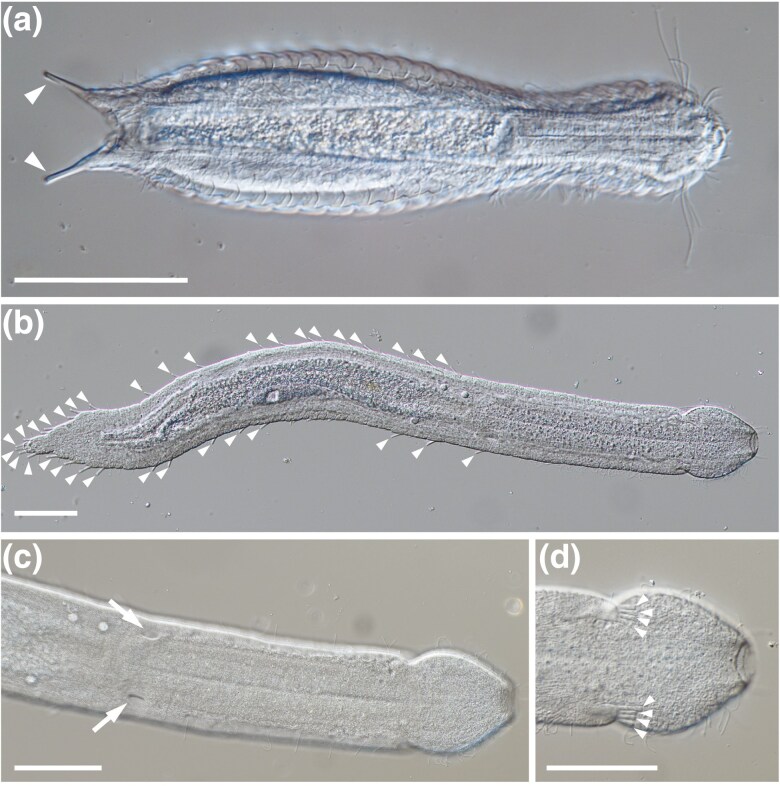
Differential interference contrast microscopy (Nomarski) images of representatives of a chaetonotidan and a macrodasyidan species showing some of the morphological differences between two orders. a) *Lepidodermella* sp. (Chaetonotida) with bottle-shaped body and two adhesive tubes situated in posterior part of the body (arrowheads). b) *Cephalodasys* sp. (Macrodasyida) with vermiform body and multiple adhesive tubes situated in the posterior part and along the lateral sides of the body (arrowheads). c and d) Details of pharyngeal pores (arrows in c) and anterior adhesive tubes (arrowheads in d) in *Cephalodasys* sp. Scale bars = 50 µm.

**Fig. 2.**
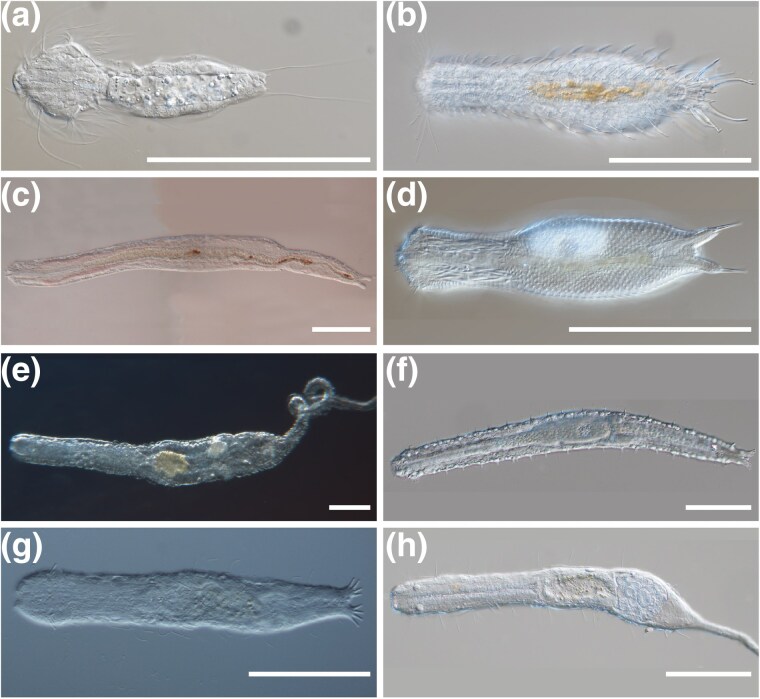
Differential interference contrast microscopy (Nomarski) images of representatives of studied gastrotrich groups. a to d) Chaetonotida; e to h) Macrodasyida. a and b) Representatives of the parthenogenetic Oiorpata clade: *Setopus* sp. and *C. neptuni* respectively. c and d) Representatives of the hermaphroditic marine *Neodasys* sp. and *X. intermedia*, respectively. e and f) Representatives of hermaphroditic species: *U. apuliensis* and *T. ambronensis*, respectively. g and h) Representatives of parthenogenetic species: *An. agadasys* and *U. viviparus*, respectively. Scale bars = 100 µm.

The first phylogenetic studies of Gastrotricha based on molecular data began to emerge in the early 2000s, primarily using partial SSU rDNA gene sequences (Todaro et al. 2003, 2006; Zrzavy 2003; Manylov et al. 2004; Paps and Riutort 2012). These reconstructions clearly divided the phylum into two well-supported clades: Macrodasyida and Chaetonotida. These groupings align well with the general morphology of the members of these orders, with macrodasyidans characterized by a vermiform body, multiple adhesive tubes, the presence of pharyngeal pores, and a pattern of an inverted Y of the cross-sectioned pharyngeal lumen, and chaetonotidans by a tenpin- or bottle-shaped body, mainly a pair of adhesive tubes, a cross-sectioned Y-shaped pharyngeal lumen, and a lack of pharyngeal pores (Kieneke and Schmidt-Rhaesa 2015; see details in Figs. 1 and 2).

The status and phylogenetic positions of several taxa remained unresolved, calling for further research (Kieneke and Schmidt-Rhaesa 2015). Subsequent phylogenetic reconstructions that incorporated multiple genes, such as rDNA SSU, LSU, and mtCOI, have provided more robust insights. However, these studies have often focused on individual taxonomic groups at various levels, from genus to order. Examples include *Heterolepidoderma* (Križanová and Vďačný 2024), *Urodasys* (Cesaretti et al. 2024), Chaetonotidae (Kånneby et al. 2013; Kolicka et al. 2020), and Macrodasyida (Todaro et al. 2012; Cesaretti et al. 2025). A recent study by Gammuto et al. (2024) contributes to this framework by providing insights into the phylogenetic relationships within the Oiorpata, a clade that encompasses both marine and freshwater Chaetonotida that primarily reproduce through parthenogenesis. Despite the numerous studies, many questions about the evolution and systematics of the involved taxa remain unanswered, which has led to ongoing debate and inquiry within the scientific community.

Mitochondrial genomes are typically rather conserved in many vertebrates and invertebrates; however, it was shown that various taxa (eg Cnidaria, Annelida, Porifera, and Mollusca) exhibit structural rearrangements (Shtolz and Mishmar 2023; Struck et al. 2023). Therefore, not only mitochondrial gene sequences but also aspects of mitochondrial genome architecture, such as gene order and structural features, can offer insights into lineage-specific evolutionary patterns (Dowling and Wolff 2023; Xiao et al. 2025).

Our study aims to investigate the molecular architecture of mitochondrial genomes in gastrotrichs across different lineages to identify potential evolutionary events linked to the biology and ecology of these minute organisms.

## Results

### Phylogeny of Gastrotrichs Based on Concatenated Mitochondrial Protein-Coding Genes

**Fig. 3.**
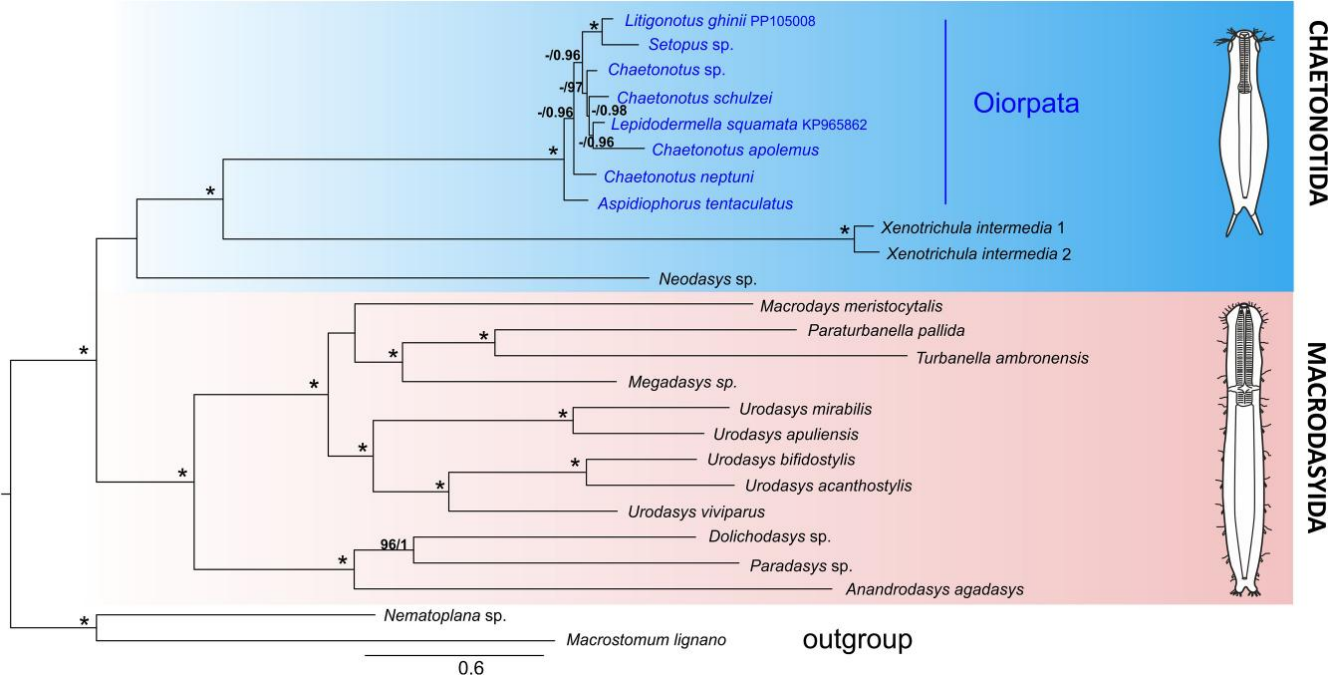
Phylogenetic tree of 22 gastrotrich species and two flatworm out-groups based on a concatenated alignment of 13 mitochondrial protein-coding genes (*cox1-3*, *cob*, *nad1-6*, *nad4L*, *atp6*, *atp8*). The topology is derived from the IQ-TREE ML analysis. Branch support values are shown as Ultrafast Bootstrap (ML) and PP (BI). Asterisks indicate 100% support for both, while values <95% are not shown. The scale bar represents substitutions per site.

We have successfully obtained and analyzed 21 gastrotrich mitochondrial genomes, of which 9 belong to Chaetonotida and 12 to Macrodasyida (Table 1). Phylogenetic analyses based on maximum likelihood (ML) and Bayesian inference (BI) approaches generated similar results, revealing two main groups, including members of Chaetonotida and Macrodasyida (Fig. 3), concordant with the previous phylogenetic analyses based on rDNA SSU gene (Paps and Riutort 2012; Bekkouche and Worsaae 2016). Within Chaetonotida, the parthenogenetic species are grouped with 100% BB (bootstrap) and posterior probability (PP) support values, forming the Oiorpata subclade as it was suggested recently (Gammuto et al. 2024). Within Oiorpata, the marine species (*Aspidiphorus tentaculatus* and *Chaetonotus neptuni*) appear as early-branching lineages within the group, suggesting a possible marine origin of the extant Oiorpata, although these nodes receive only 96% PP support. Two marine hermaphroditic taxa, *Xenotrichula* and *Neodasys*, appear as basal lineages to Oiorpata. While *Xenotrichula* is branching with the Oiorpata with 100% BB and PP support, the position of *Neodasys* still remains unresolved (Fig. 3). The macrodasyidan clade in turn is monophyletic with 100% BB and PP support, where two well-supported subclades can be observed: (i) represented by two members of the family Cephalodasyidae, namely *Paradasys* sp. and *Dolichodasys* sp., together with *Anandrodasys agadasys*, a representative of the Redudasyidae and (ii) represented by all remaining taxa (ie *Turbanella*, *Paraturbanella*, *Megadasys*, *Urodasys*, and *Macrodasys*). Within this last subclade: (i) all members belonging to genus *Urodasys* clustered together supporting the monophyly of the genus; (ii) two members from the family Turbanellidae, such as *Turbanella ambronensis* and *Paraturbanella pallida*, together with *Megadasys* sp. formed a well-supported subclade; and (iii) the position of *Macrodasys meristocytalis* is weakly supported, with low BB and PP values preventing confident placement within the subclade. Additionally, we obtained a similar tree when analyzing the same dataset but excluding *atp6* and *atp8* (Supplementary Material S1), since these genes were not detected in several mitogenomes (see below). This confirms that omitting these genes neither alters the overall tree topology nor improves support for the putative nodes.

**Table 1:** The comparison of mitochondrial genome length, gene number, GC content, repeats, and transcriptional order through studied gastrotrich lineages

	Length of mtDNA (bp)	Number of PCGs	Number of tRNA	GC %	Number of tandem repeats	Transcriptional direction of the genes
Species (Chaetonotida)						
*Aspidiophorus tentaculatus*	14,547	13	22	38	1	Unidirectional with exception trnT, trnD, trnP
*Chaetonotus* sp.	14,426	13	22	40	0	Unidirectional with exception trnT, trnD, trnP
*Chaetonotus neptuni*	14,585	13	22	37	1	Unidirectional with exception trnT, trnD, trnP
*Chaetonotus schultzei*	14,503	13	22	40	1	Unidirectional with exception trnT, trnD, trnP
*Chaetonotus apolemmus*	14,597	13	22	40	0	Unidirectional with exception trnT, trnD, trnP
*Lepidodermella squamata*	14,558	13	22	40	1	Unidirectional with exception trnT, trnD, trnP
*Litigonotus ghinii*	14,384	13	22	42	0	Unidirectional with exception trnT, trnD, trnP
*Setopus* sp.	14,495	13	22	42	0	Unidirectional with exception trnT, trnD, trnP
*Xenotrichula intermedia 1*	15,095	12	22	31	0	Unidirectional with no exception
*Xenotrichula intermedia 2*	14,919	12	22	31	0	Unidirectional with no exception
*Neodasys* sp.	14,156	12	22	40	0	Unidirectional with no exception
Species (Macrodasyida)						
*Anandrodasys agadasys*	16,207	11	17	29	2	Not unidirectional, cox1, cox2, cox3, cob, nad2, nad3, nad5, nad6, rrnL and trnH, S1, Y, R, F, W have opposite direction
*Dolichodasys* sp.	15,893	11	22	26	0	Not unidirectional, cox1, cox2, cox3, cob, nad1, nad2, nad4, nad4L nad6, rrnL and trnP, F, S1, S2, E, A, T, I, V, L1, C, M have opposite direction
*Macrodasys meristocytalis*	14,402	11	21	29	9	Not unidirectional, cox1, cox2, nad1, nad5, nad4l, rrnS, and trnT, E, Q, L1, P, S1, C have opposite direction
*Megadasys* sp.	14,487	11	21	24	5	Not unidirectional, cox1, cox3, nad2, nad6 rrnS, rrnL and trnQ, N, L1, D, C, l, S2, K, R, P, F, L2, S1, W have opposite direction
*Paradasys* sp.	12,838	11	17	25	2	Not unidirectional, cox1-0, nad4, nad4L, nad5, nad6 and trnV, M, A, P, S2; R, G, Y have opposite direction
*Paraturbanella pallida*	14,981	11	22	20	7	Not unidirectional, cox3, cox2, nad1-3, rrnL, rrnS and trnS1, S2, D, F, Y, K, S2; M, G, L2; P have opposite direction
*Turbanella ambronensis*	14,297	11	17	46	1	Not unidirectional, but only for tRNAs such as trnQ, N, L2, T, K, H, F have opposite direction
*Urodasys bifidostylis*	15,624	11	19	21	8	Not unidirectional, cox3, cox2, nad6, 4L, nad5, rrnL and trnT, W, S2, Y, K, l, M, R, A, Q have opposite direction
*Urodasys mirabilis*	19,009	11	22	31	2	Not unidirectional, cox1, cox2-1/0, cox3, nad4L, nad2, nad4, nad5, rrnS, rrnL and trnP, G, Y, D, E, I, M, T, C, W, S1 have opposite direction
*Urodasys apuliensis*	18,723	11	20	22	4	Not unidirectional, cob, nad1, nad4-1, nad4-0, nad6, and trnE, L1, G, H, Q, N, V, S2, D, F, R, K have opposite direction
*Urodasys acanthostylis*	15,508	11	19	22	2	Not unidirectional, cox1, cox2, nad1, nad3, nad4, nad5, nad6, rrnL and trnP, G, F, H, V, W, S2, T, Y, K, D, I, M, S1 have opposite direction
*Urodasys viviparus*	13,219	11	22	20	8	Not unidirectional, cox1, cox2, cox3, cob, nad1, nad3, nad5, nad6, rrnL, rrnS, and trnT, Y, A, I, D, L1, W, R, S1, Q, M, V have opposite direction

### General Structure of Gastrotrich Mitogenomes

**Fig. 4.**
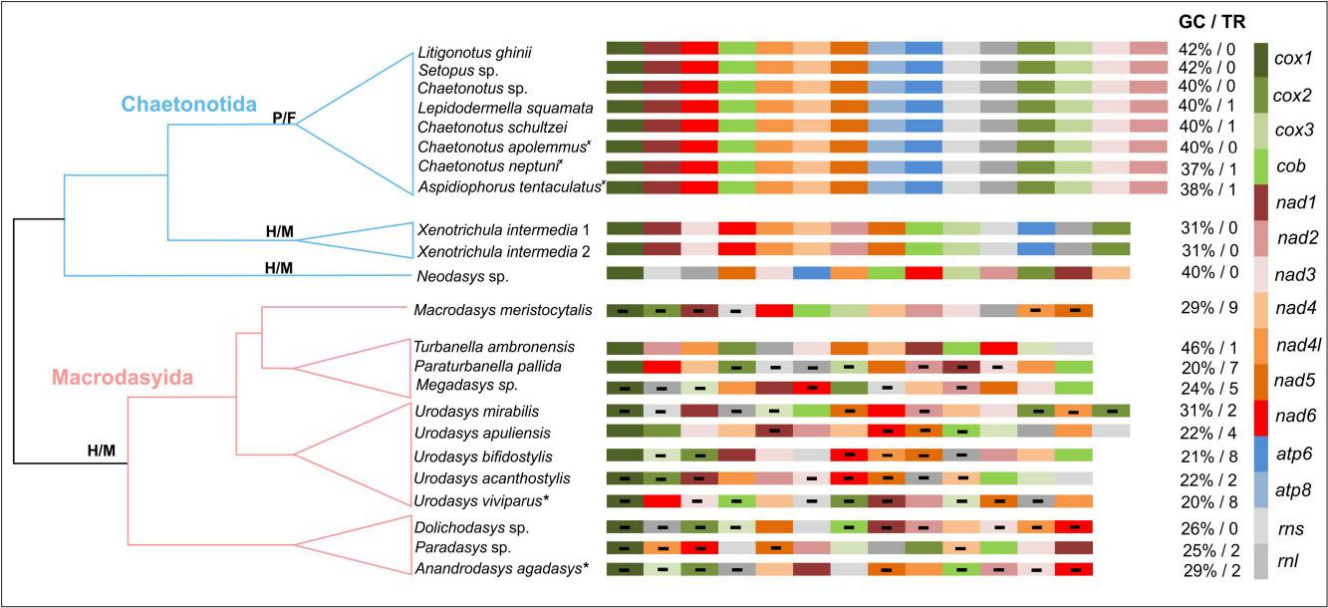
Mitochondrial protein-coding and ribosomal gene order mapped to schematic phylogenetic relationships of the studied gastrotrich species. Abbreviations on the nodes indicate: P/F-parthenogenetic freshwater species, H/M-hermaphroditic marine species. Asterisks indicate exception for *U. viviparus* and *An. agadasys* which are parthenogenetic. Crosses indicate exception for *C. apolemmus*, *C. neptuni*, and *A. tentaculatus* which are marine. Gene boxes are color coded by gene name. “−” symbol within a box indicates that the gene is located on the minus strand, while empty boxes represent genes on the plus strand. GC/TR values denote the GC content (%) of the mitochondrial genome and the number of tandem repeats identified.

The complete mitochondrial genomes of the studied gastrotrichs are single circular molecules ranging from 13 to 19 kb in length (Table 1). They consist of 11 to 13 protein-coding genes, 17 to 22 tRNA genes, and 2 rRNA genes. The structural analyses revealed a clear distinction between members of the two orders in terms of mitogenome length, protein-coding gene number and order, GC content, the direction of encoded genes, tandem repeat numbers, and codon usage preferences. Additionally, we observed conserved mitogenome structures within the Oiorpata group (parthenogenetic chaetonotidans), whereas macrodasyidan species displayed considerable variability (Fig. 4). Conserved structural patterns were noted to some extent in hermaphroditic marine chaetonotidans (eg the number of protein-coding genes is 12 vs. 13 in Oiorpata, *atp8* being the missing gene); however, the limited number of samples in this group precludes definitive conclusions. Our analyses showed that chaetonotidan mitogenomes are quite conserved in terms of mitogenome size (14,156 to 15,103 bp), with Oiorpata species ranging from 14,384 to 14,558 bp. In contrast, the mitogenome length is more variable in Macrodasyida, spanning from 13,340 to 19,008 bp (Table 1). This high range in the length of mtDNA is mainly due to two outliers, *Urodasys apuliensis* and *Urodasys mirabilis*, for which a possible gene duplication event was detected (see details below). Base composition analyses indicated that chaetonotidan mtDNA has higher GC content (37% to 42%, except for *Xenotrichula intermedia* at 31%) compared with macrodasyidan mtDNA (GC = 20% to 29%, except for *T. ambronensis* at 46%) (Table 1, Supplementary Material S2). Tandem repeat analyses showed a similar pattern, with no or at most one tandem repeat found in chaetonotidans, while these repeats are more common in observed macrodasyidan species, ranging from 1 to 9 with variable copy numbers (Table 1, Supplementary Material S3). Statistical comparisons of GC content (Student's *t*-test) and tandem repeat abundance (Mann–Whitney *U* test) revealed significant differences in mitochondrial genome composition and architecture between the two orders (see Materials and Methods for details). GC content was significantly higher in Chaetonotida compared with Macrodasyida (*t*-test, *P* = 3.82 × 10⁻^5^). Similarly, tandem repeat content differed significantly between the groups (Mann–Whitney *U* test, *P* = 3.87 × 10⁻^4^). Correlation analyses indicated a strong negative association between GC content and tandem repeat abundance (Spearman's *r_s_* = −0.729, *P* = 3.94 × 10⁻^5^). We did not observe gene duplication events, with the exception of two representatives of early-branching lineages of *Urodasys* species: *U. mirabilis* (with duplication of the *cox2* gene) and *U. apuliensis* (with duplication of the *nad4* gene; Fig. 4). Re-sequencing the same specimens with Oxford nanopore technology confirmed these duplications. Additionally, while one of the duplicated sequences in both species showed higher variability, both the crystal structure and specific core regions remained highly conserved across all sequences, supporting the existence of a true duplication rather than a bioinformatic artifact.

### Protein-Coding Gene Number, Order, and Transcriptional Direction

**Fig. 5.**
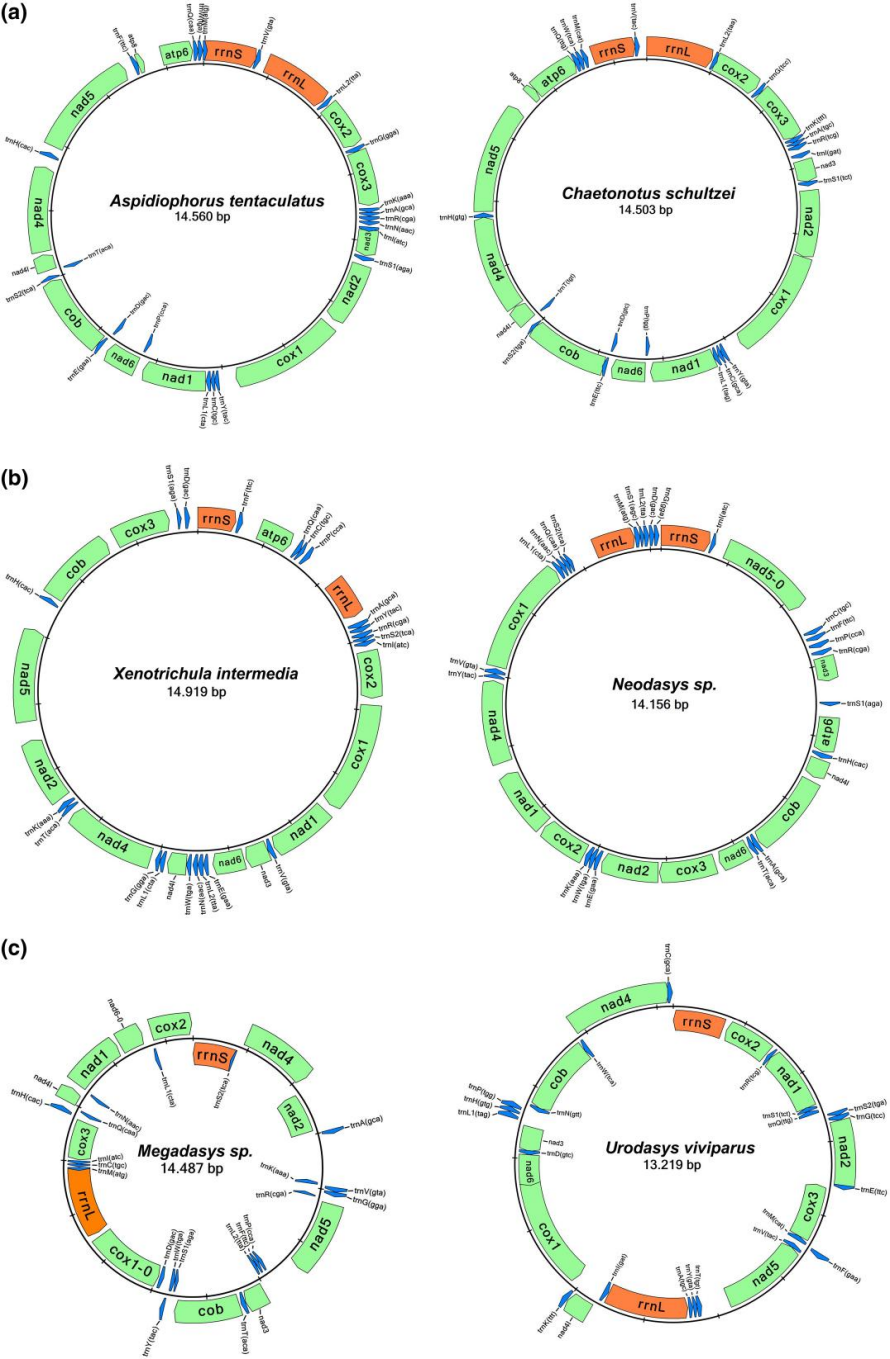
Comparison of gene transcriptional direction in representatives of the different gastrotrich linages. a) Almost unidirectional transcription with exception of trnT, trnD, trNP in the members of Oiorpata clade, b) complete unidirectional transcription in the members of marine hermaphroditic chaetonotidans, and c) mixed directional transcription in the macrodasyidans. Protein-coding genes are in green, ribosomal genes are in orange, tRNAs are in blue.

We observed complete synteny (same gene order) in parthenogenetic chaetonotidans (Oiorpata group) with 13 protein-coding genes (*cox1-3*, *cob*, *nad1-6, nad4l*, *atp6*, *atp8*; Fig. 4) and unidirectional transcription, where all genes have the same direction (“+” strand as annotated by MITOS2, which corresponds to the 5′→3′ direction of the reference scaffold), except for *trnT*, *trnD*, and *trnP* (Fig. 5). The mitochondrial architecture differs slightly in exclusively marine and hermaphroditic chaetonotidans (representatives of the families Neodasyidae and Xenotrichulidae), which have 12 protein-coding genes (*atp8* was not detected). The gene order in these species differs from other chaetonotidans, but all genes are transcribed in the same direction. In contrast, macrodasyidans have only 11 protein-coding genes, with *atp6* and *atp8* absent, and their gene order is highly variable. The transcriptional direction of macrodasyidan mitochondrial genes also varies widely, with no conserved patterns even at the genus level (eg no common pattern was observed in five species of the genus *Urodasys* included in this study; Table 2). We also observed a significant negative correlation between GC content and transcriptional directionality (Spearman's *r_s_* = −0.749, *P* = 1.94 × 10⁻^5^), suggesting that mitogenomes with higher GC content tend to have more unidirectional gene orientation.

**Table 2:** Codon usage preferences in gastrotrich species

Species	Phe	Leu	Ile	Met	Val	Ser	Pro	Thr	Ala	Tyr	His
*Aspidiophorus tentaculatus*	F1	L1	I1	M2	V1	S1	P1	T1	A1	Y1	H2
*Litigonotus ghinii*	F1	L3	I1	M2	V1	S1	P1	T1	A1	Y1	H2
*Chaetonotus* sp.	F1	L3	I1	M2	V1	S1	P1	T1	A1	Y1	H1
*Chaetonotus neptuni*	F1	L1	I1	M2	V1	S1	P1	T1	A1	Y1	H1
*Chaetonotus schultzei*	F1	L3	I1	M2	V1	S1	P1	T1	A1	Y1	H1
*Chaetonotus apolemmus*	F1	L3	I1	M2	V1	S1	P1	T1	A1	Y1	H1
*Lepidodermela squamata*	F1	L3	I1	M2	V1	S1	P1	T1	A1	Y1	H2
*Setopus* sp.	F1	L1	I1	M2	V1	S1	P1	T1	A1	Y1	H2
*Xenotrichula intermedia 1*	F1	L1	I1	M1	V1	S1	P1	T1	A1	Y1	H2
*Xenotrichula intermedia 2*	F1	L1	I1	M1	V1	S1	P1	T1	A1	Y1	H2
*Neodasys sp.*	F1	L1	I1	M1	V1	S1	P1	T1	A2	Y1	H2
*Anandrodasys agadasys*	F1	L1	I1	M1	V1	S1	P1	T1	A1	Y1	H1
*Dolichodasys* sp.	F1	L1	I1	M1	V1	S1	P3	T1	A1	Y1	H1
*Macrodasys meristocytalis*	F1	L1	I1	M1	V1	S1	P1	T1	A1	Y1	H1
*Megadasys* sp.	F1	L1	I1	M1	V1	S1	P1	T1	A1	Y1	H1
*Paradasys* sp.	F1	L1	I1	M1	V1	S1	P1	T1	A1	Y1	H1
*Paraturbanella pallida*	F1	L1	I1	M1	V1	S1	P1	T1	A1	Y1	H1
*Turbanella ambronensis*	F1	L3	I1	M2	V1	S1	P2	T2	A1	Y2	H2
*Urodasys bifidostylis*	F1	L1	I1	M1	V1	S1	P1	T1	A1	Y1	H1
*Urodasys mirabilis*	F1	L1	I1	M1	V1	S1	P1	T1	A1	Y1	H1
*Urodasys apuliensis*	F1	L1	I1	M1	V1	S1	P1	T1	A1	Y1	H1
*Urodasys acanthostylis*	F1	L1	I1	M1	V1	S1	P1	T1	A1	Y1	H1
*Urodasys viviparus*	F1	L1	I1	M1	V1	S1	P1	T1	A1	Y1	H1
	UUU(F1)	UUA(L1)	AUU(I1)	AUA(M1)	GUU(V1)	UCU(S1)	CCU(P1)	ACU(T1)	GCU(A1)	UAU(Y1)	CAU(H1)
	UUC(F2)	UUG(L2)	AUC(I2)	AUG(M2)	GUC(V2)	UCC(S2)	CCC(P2)	ACC(T2)	GCC(A2)	UAC(Y2)	CAC(H2)
	…	CUU(L3)	…	…	GUA(V3)	UCA(S3)	CCA(P3)	ACA(T3)	GCA(A3)	…	…
	…	CUC(L4)	…	…	GUG(V4)	UCG(S4)	CCG(P4)	ACG(T4)	GCG(A4)	…	…
	…	CUA(L5)	…	…	…	…	…	…	…	…	…
	…	CUG(L6)	…	…	…	…	…	…	…	…	…

Species	Gly	Asn	Lys	Asp	Glu	Arg	Ser	Gly	Cys	Trp
*Aspidiophorus tentaculatus*	Q2	N1	K1	D1	E1	R3	S3	G1	C1	W1
*Litigonotus ghinii*	Q2	N2	K1	D2	E1	R3	S3	G4	C2	W1
*Chaetonotus* sp.	Q2	N2	K1	D2	E2	R3	S3	G1	C2	W2
*Chaetonotus neptuni*	Q2	N1	K1	D1	E2	R3	S3	G1	C1	W1
*Chaetonotus schultzei*	Q2	N1	K1	D1	E2	R4	S1	G1	C1	W2
*Chaetonotus apolemmus*	Q1	N1	K1	D1	E1	R4	S4	G1	C1	W2
*Lepidodermela squamata*	Q2	N2	K1	D2	^a^	R3	S3	G1	C2	W2
*Setopus* sp.	Q2	N2	K1	D1	E1	^a^	S4	G4	C1	W2
*Xenotrichula intermedia 1*	Q2	N2	K1	D1	E1	^a^	S4	G4	C1	W2
*Xenotrichula intermedia 2*	Q2	N2	K1	D1	E1	^a^	S4	G4	C1	W2
*Neodasys sp.*	Q1	N1	K2	D2	E2	R3	S4	G4	C1	W2
*Anandrodasys agadasys*	Q1	N1	K2	D1	E2	R1	S1	G1	C1	W2
*Dolichodasys* sp.	Q1	N1	K1	D1	E1	R3	S1	G1	C1	W1
*Macrodasys meristocytalis*	Q1	N1	K1	D1	E1	R1	S3	G1	C1	W1
*Megadasys* sp.	Q1	N1	K1	D1	E1	R3	S3	G3	C1	W1
*Paradasys* sp.	Q1	N1	K1	D1	E1	R3	S3	G3	C1	W1
*Paraturbanella pallida*	Q1	N1	K1	D1	E1	R3	S3	G3	C1	W1
*Turbanella ambronensis*	Q2	N2	K2	D2	E2	R1	S4	G4	C1	W2
*Urodasys bifidostylis*	Q1	N1	K1	D1	E1	R3	S3	G3	C1	W1
*Urodasys mirabilis*	Q1	N1	K1	D1	E1	R1	S3	G1	C1	W1
*Urodasys apuliensis*	Q1	N1	K1	D1	E1	R1	S3	G1	C1	W1
*Urodasys acanthostylis*	Q1	N1	K1	D1	E1	R1	S3	G3	C1	W1
*Urodasys viviparus*	Q1	N1	K1	D1	E1	R3	S3	G3	C1	W1
	CAA(Q1)	AAU(N1)	AAA(K1)	GAU(D1)	GAA(E1)	CGU(R1)	AGU(S1)	GGU(G1)	UGU(C1)	UGA(W1)
	CAG(Q2)	AAC(N2)	AAG(K2)	GAC(D2)	GAG(E2)	CGC(R2)	AGC(S2)	GGC(G2)	UGC(C2)	UGG(W2)
	…	…	…	…	…	CGA(R3)	AGA(S3)	GGA(G3)	…	…
	…	…	…	…	…	CGG(R4)	AGG(S4)	GGG(G4)	…	…

^a^When codon usage preference is equal.

Additional analyses were conducted to search for the *atp* genes. The *atp8* gene was not recovered by MITOS2 in hermaphroditic marine chaetonotidan mtDNA, and the *atp6* and *atp8* genes were not recovered in macrodasyidan mtDNA as well. We identified *atp6* candidates in all studied macrodasyidan nuclear contigs, with the exception of *Urodasys bifidostylis* and *Dolychodasys* sp. (Supplementary Material S4), likely due to the lower quality of the assembly, in which the specific contig containing *atp6* may have been lost. Notably, in *T. ambronensis*, *atp6* was found on two distinct scaffolds. In all species, MITOS2 identified *atp6* as an isolated gene, without any neighboring mitochondrial genes (that instead should be located in a unique contig). Exonerate also produced similar and consistent results, further supporting the nuclear localization of *atp6*. The comparison of putative *atp6* sequences against the nonredundant protein database (nr) using Blastx, confirming their identity as *atp6*. Additionally, nuclear-localized candidate *atp6* sequences were translated using the standard nuclear genetic code and analyzed for mitochondrial targeting signals. Most sequences were compatible with the standard code and exhibited protein lengths (∼155 amino acids) comparable to those found in Chaetonotida, suggesting potential functionality. However, in four species (*U. mirabilis*, *Urodasys viviparus*, *U. apuliensis*, and *T. ambronensis*), premature stop codons were detected under standard code translation, which may reflect assembly artifacts, pseudogenization, or alternative coding interpretations. TargetP (Emanuelsson et al. 2000) predicted putative mitochondrial targeting signals only in *U. mirabilis*, *T. ambronensis*, and *P. pallida* indicating a potential functional relocation of *atp6* in these species, pending further validation. Conversely, *atp8* was not detected in any assembly, likely due to its short length, making it difficult to identify.

### Codon Usage Analysis

Codon usage analysis revealed conserved patterns for some amino acids across all studied gastrotrichs. For example, in the surveyed species, phenylalanine is most commonly encoded by UUU, isoleucine by AUU, valine by GUU, and serine by UCU. Additionally, lineage-specific patterns were observed for other amino acids. For instance, in members of the Oiorpata group, the most commonly used codon is AUG for methionine while all other examined gastrotrichs prefer AUA (except for macrodasyidan species *T. ambronensis* which prefers AUG, and has a higher CG content of 46%). Oiorpata members also have a preference of CCU for proline, ACU for threonine, and AAA for lysine (Table 2).

## Discussion

While mitochondrial structure and function vary across eukaryotes, mitogenome content tends to be conserved in major groups such as metazoans (Shtolz and Mishmar 2023). Yet, several lineages have undergone gene rearrangements, expansions, losses, or even genome fragmentation often associated with factors like extreme habitats, high metabolic rates, short generation times, or parasitic lifestyles (Yahalomi et al. 2020; Feng et al. 2022; Struck et al. 2023). Understanding these patterns provides a valuable framework for interpreting the mitogenomic diversity observed in Gastrotricha.

Gastrotrichs, a fascinating group of microscopic metazoans, have diversified in marine and freshwater habitats, adopting various lifestyles and reproductive strategies, which seem related to variations in their mitochondrial genomes. Our phylogenetic analyses separated the studied gastrotrich species into two groups corresponding to the currently recognized two orders, reflecting well their morphology, reproductive biology, and lifestyle. For instance, in the chaetonotidan group, the Oiorpata comprises parthenogenetic species, while the hermaphroditic species branch as basal lineages to Oiorpata (Fig. 3). Within Oiorpata, two marine species appear as early-diverging clades, indicating a possible marine origin of the group, providing support to the evolutionary scenario hypothesized by previous phylogenetic analyses based on nuclear ribosomal genes and a denser taxonomic sampling (Kånneby et al. 2013; Bekkouche and Worsaae 2016; Saponi and Todaro 2024; Minowa et al. 2025).

Kolicka et al. (2020) have challenged this scenario by proposing that the basal position of certain marine species along the Oiorpata evolutionary branch, as indicated by phylogenetic analyses based on nuclear genes (and mtCOI), might be an artifact resulting from a long-branch attraction (LBA) effect (Bergsten 2005). The LBA may lead to these marine species appearing closer to other unrelated, early-diverging, long-branched chaetonotidan clades, such as the Xenotrichulidae and Muselliferidae (Kånneby et al. 2013; Bekkouche and Worsaae 2016). Indeed, in all previous phylogenetic studies involving marine species of the genus *Aspidiophorus* and the subgenus *Schizochaetonotus* (genus *Chaetonotus*), the length of their branches is significantly greater (ie about three times longer) than that of other Oiorpata, as noted by Kolicka et al. (2020). Their branch lengths are also somewhat comparable to the long branches of Xenotrichulidae. When *Aspidiophorus* and *Schizochaetonotus* species are excluded from the analyses, the phylogenetic scenario changes, revealing other taxa like *Bifidochaetonotus* as early-divergent lineages within the Oiorpata (Kolicka et al. 2020). This new scenario suggests that the extant Oiorpata likely originated in freshwater environments, followed by a secondary invasion of the sea by all the marine species within this group.

Our phylogenetic analyses, similar to the studies mentioned earlier, show Xenotrichulidae as the sister group to Oiorpata, with the marine species *A. tentaculatus* and *C. neptuni* (subgenus *Schizochaetonotus*) representing the earliest diverging lineages of the latter group. Moreover, in our analyses, we found that the branch lengths leading to *A. tentaculatus* and *C. neptuni* are comparable to those of other Oiorpata species. This suggests that their basal position is not a result of LBA, which challenges the hypothesis proposed by Kolicka et al. (2020) while revitalizing the evolutionary scenario envisioned by earlier studies that posited a marine origin for extant Oiorpata. Furthermore, in our study, the derived position of *Chaetonotus apolemmus* suggests that a secondary invasion of the marine environment has occurred, but this phenomenon applies only to some lineages, as previously found by the seminal work of Kånneby et al. (2013). Our study also provides an indication of *Neodasys* being a chaetonotidan branch, implied by the current classification, although with low statistical support (Fig. 3).

The classification of *Neodasys* has significantly evolved over time, highlighting the challenges in achieving consistent hypotheses regarding phylogenetic relationships. Originally established by Remane (1927) within the Macrodasyida, this intriguing genus faced a transformative reclassification due to meticulous histological analyses of the pharynx in *Neodasys chaetonotoideus*. These studies revealed a striking morphological feature: the pharyngeal lumen, when viewed in cross-section, forms an inverted Y shape. This distinctive characteristic aligns with the hallmark structure found in all Chaetonotida, ultimately justifying its important shift into this category (Remane 1936). The distinct differences in body shape, reproductive biology, and adhesive apparatus of *Neodasys* compared with other chaetonotidans led Hondt (1971) to establish two suborders: Multitubulatina, which includes *Neodasys*, and Paucitubulatina, which encompasses the other chaetonotidans. This classification emphasizes the clear disparities between *Neodasys* and the rest of the chaetonotidans (Ruppert 1991). Since then, *Neodasys*, which currently comprises three described species (Saponi and Todaro 2024), has been included in several phylogenetic reconstructions based on morphological or molecular datasets (Todaro et al. 2003, 2006; Marotta et al. 2005; Petrov et al. 2007; Kieneke et al. 2008; Paps and Riutort 2012); however, it has never been convincingly assigned to either order. Like previous studies, our phylogenetic analyses based on 13 mitochondrial protein-coding genes failed to consistently determine the position of *Neodasys*, highlighting the urgent need of analyses based on a broader sampling of species and genes.

Our results show that structures of the mtDNA in gastrotrichs are order specific; for example, 12 to 13 PCGs in Chaetonotida versus 11 PCGs in Macrodasyida, high GC content in Chaetonotida versus low GC content in Macrodasyida, 0 to 1 tandem repeats in Chetonotida versus >1 repeats in Macrodasyida, almost or exclusively unidirectional mtDNA in Chaetonotida versus nonunidirectional mtDNA Macrodasyida. Notably, the gene synteny (the same gene order) is perfectly conserved among all investigated members of Oiorpata, even among those from different genera and families. Conversely, the situation is quite different in the order Macrodasyida, where there is no synteny (the gene order is not conserved) even among members of the same genus, as illustrated by *Urodasys*. These observed structural variations of the gastrotrich mitogenomes may be related to several factors, with mtDNA stability (eg less prone to mutations) being perhaps the most important. Chaetonotidan mtDNA appears to be more stable and less prone to mutations than macrodasyidan mtDNA, likely due to its higher GC content and, more notably, fewer tandem repeats (Yakovchuk et al. 2006; Chen and Skylaris 2021; Table 1). It is possible that this stability facilitated the conservation of the gene order (Fig. 4) and gene transcriptional directionality (Table 1, Fig. 5) in chaetonotidans, preserving their mtDNA from rapid mutation as it was suggested in other organisms (Nguyen et al. 2020). In contrast, macrodasyidan mtDNA is prone to higher mutation rates, because it is characterized by lower GC content, a higher number of tandem repeats, variable gene order, and variable gene directionality. Indeed, it was shown that macrodayidans exhibit a generalized high mutation rate, as indicated by previous phylogenetic analyses based on nuclear and mitochondrial genes. These studies reveal that members of this lineage have higher nucleotide and amino acid substitution rates and longer branch lengths, when compared with chaetonotidans (Bekkouche and Worsaae 2016). Increased nucleotide substitution rates may lead to increased gene rearrangement rates, as proposed in other metazoans (eg for insects, as discussed in Shao et al. 2003, or for annelid worms, as discussed in Struck et al. 2023). Codon usage bias (CUB) frequencies may influence mitogenome structural variation as well. For instance, CUB analysis revealed that CUB patterns may reflect mutational bias and natural selection, as suggested for reptiles (Montaña-Lozano et al. 2023). Lastly, tRNA genes can also play a crucial role in mitochondrial genome rearrangements affecting its architecture (Prada and Boore 2019; Moreno-Carmona et al. 2021). Indeed, we can notice that while the number of tRNAs is conserved in chaetonotidans (22), it is highly variable in macrodasyidans (17 to 22). We are not, however, excluding that some tRNA might not be detected with the algorithms integrated in MITOS2 or with manual alignment due to their extreme divergence in macrodasyidans.

Interestingly, *T. ambronensis* appears to be an exception among studied macrodasyidans, exhibiting a relatively high GC content (46%) compared with other macrodasyidan species (20% to 31%). Notably, *T. ambronensis* also diverges in gene directionality: only six tRNAs are encoded on the reverse (“−”) strand, while all other genes are located on the forward (“+”) strand, which corresponds to the 5′→3′ direction of the reference scaffold. In contrast, most macrodasyidan species show a more balanced distribution of genes across both strands. A similar trend emerges from CUB analysis: for instance, *T. ambronensis* shows a codon preference more closely aligned with chaetonotidans than with other macrodasyidans (Table 2). Indeed, all observed chaetonotidan species exhibit high GC content and predominantly, if not exclusively, unidirectional mitochondrial genomes.

These observations suggest a possible link between GC content and CUB in mtDNA. Although gene directionality in *T. ambronensis* remains distinct from chaetonotidan species, which typically exhibit almost unidirectional mitochondrial genomes, it shows a partial resemblance to *Oiorpata*, where only three tRNAs are encoded on the (“−”) strand. This contrasts with most macrodasyidan species, in which a substantial portion of protein-coding genes are located on the (“−”) strand. These intermediate features in *T. ambronensis* may reflect lineage-specific variation or partial convergence in mitochondrial genome organization. Alternatively, it is possible that these features represent ancestral characteristics retained in this species but independently lost or modified in other lineages due to relaxed selective pressure or lineage-specific adaptation. However, this remains a working hypothesis, and additional genomic data from T. ambronensis is needed to verify and clarify these patterns.

We also tried to explore potential connections between mitochondrial genome architecture and the lifestyle of the studied species, as inferred from their morphology and anatomy. Although we did not observe a direct correlation between mitogenome structure and marine versus freshwater habitats (eg both marine and freshwater species of *Oiorpata* exhibit conserved mitogenome features), certain anatomical and metabolic traits may still influence mitochondrial organization. For example, dorsoventral muscles are typically found in flat, hermaphroditic taxa inhabiting sandy marine environments (eg Xenotrichulidae), but are generally absent in flask-shaped, benthic freshwater taxa (eg Chaetonotidae). It has been proposed that these muscles originated in marine interstitial hermaphrodites and were progressively reduced or lost as species transitioned to epibenthic or periphytic lifestyles and parthenogenetic reproduction (Leasi et al. 2006; Leasi and Todaro 2008). These anatomical changes may reflect shifts in bioenergetic demands, potentially influencing mitogenome evolution. Supporting the link between mitogenome structure and anatomical traits of the organism, we observed gene duplications in two early-branching species of *Urodasys*: *U. mirabilis* (cox2 duplication) and *U. apuliensis* (nad4 duplication). These species also differ from other *Urodasys* taxa in reproductive morphology, notably lacking accessory copulatory organs (Cesaretti et al. 2024), which may likewise point to distinct energetic or developmental requirements. However, it is challenging to attribute the structural variability of the macrodasyidans' mitogenome to specific factors. From a biological standpoint, a notable difference between chaetonotidans from the Oiorpata group and macrodasyidans lies in their reproductive methods: these chaetonotidans reproduce through apomictic parthenogenesis, while macrodasyidans reproduce via cross-fertilization (Kieneke and Schmidt-Rhaesa 2015; Cesaretti et al. 2024; Gammuto et al. 2024). While intriguing, any potential association between reproductive mode and the absence or nuclear transfer of *atp6* and *atp8* genes remains speculative and requires further investigation, particularly given that most metazoan mitochondrial genomes retain these genes regardless of reproductive strategy.

In this highly uncertain framework, we outline three nonmutually exclusive scenarios regarding the evolution of the mitochondrial genome in Gastrotricha. The most parsimonious explanation is genetic drift, which likely underlies the independent retention, loss, or transfer of ATP synthase genes across lineages. At the same time, we note two additional possibilities that may warrant future investigation. (i) Within the mitogenome evolution module suggested by Kelly (2021), the loss or transfer of mitochondrial genes to the nucleus could be associated with bioenergetic demands in hermaphroditic macrodasyidans and marine hermaphroditic chaetonotidans, although this remains highly tentative. (ii) The retention of ATP synthase-related genes in parthenogenetic chaetonotidans could be consistent with lineage-specific regulatory adaptations (eg mitochondrial stress responses or specialized transcriptional or translational mechanisms during evolution Allen 2015; Casanova et al. 2023; Butenko et al. 2024), but this hypothesis requires further genomic data to evaluate. Additionally, although we identified *atp6* gene blast hits in the nuclear nodes of nearly all macrodasyidans and no hits for *atp8*, we cannot rule out the possibility that these genes remain undetected in other nuclear datasets (Kohn et al. 2012). This limitation may stem from challenges in assembly and annotation in Gastrotricha, as seen in related taxa such as Platyhelminthes (Shimada et al. 2023).

Practically nothing is known about the metabolic pathways of gastrotrichs, although a few hypotheses have been suggested in the past, highlighting the possibility of anaerobiosis in some species that occur deeper in the sediments (Boaden 1974, 1985; Todaro et al. 2000; Balsamo et al. 2007). While adaptation to anoxic conditions may contribute to accelerated mitochondrial genome remodeling, the directionality and mechanisms of such changes remain complex and potentially lineage-specific. Our results suggest that once an evolutionary path has been taken, it is often difficult to revert back to a previous state. This is exemplified by the macrodasyidan species *U. viviparus* and *An. agadasys*, which are parthenogenetic and still maintain a reduced number of mitochondrial genes. Although a direct link between reproductive mode and the retention of specific mitochondrial genes cannot be definitively established, the persistence of this genomic pattern raises intriguing questions about the evolutionary inertia of organelle architecture and the interplay between reproductive strategy, ecology, and mitochondrial function.

## Conclusions

In conclusion, our observations suggest the following tentative hypotheses:

1. Mitogenome conservation within Oiorpata and structural variability among Macrodasyida may be influenced by genomic features such as lower GC content and elevated tandem repeats, potentially contributing to reduced DNA stability in Macrodasyida.
2. Structural differences in mitochondrial genomes may coincide with lineage-specific aspects of reproductive biology in gastrotrichs; however, these associations remain speculative and require further investigation. For now, the genetic drift remains the most parsimonious explanation, especially given the limitations of phylogenetic nonindependence.
3. Other factors that may play a role in shaping mitochondrial genome architecture may include lineage-specific codon usage patterns, tRNA rearrangements, elevated nucleotide substitution rates during evolution, and anatomical or metabolic adaptations associated with different lifestyles.

Together, these results highlight a striking separation in mitogenome evolution, from rigid order to radical variation across the two principal lineages of Gastrotricha. They underscore the importance of lineage-specific factors in shaping mitochondrial genome architecture and demonstrate how expanded taxonomic sampling can reveal unexpected complexity in genome evolution across Metazoa.

Additionally, beyond these biological insights, our study offers two practical contributions to the field: (i) the generation of 21 new mitochondrial genomes, which is important in advancing the field of mitochondrial genetics, and (ii) the successful demonstration of the whole-genome amplification (WGA) and Blobology pipeline (see details in Materials and Methods) as an effective approach for obtaining multiple sequences from single microeukaryotes in gastrotrich studies. This achievement is particularly valuable when working with rare and old specimens containing limited genomic material. Although the focus of this study was restricted to retrieving mitochondrial genomes, the pipeline holds great potential for broader applications. It can be utilized in future studies to recover hundreds of genes, enabling research in multigene phylogeny, functional genomics, and comparative genomics.

## Materials and Methods

### Species Selection

For comparison purposes, we chose 10 chaetonotidan and 12 macrodasyidan species with variable ecological adaptations (marine vs. freshwater) and reproductive modalities (hermaphroditic vs. parthenogenetic). Two of these species, both freshwater belonging to the order Chaetonotida, already have published mitochondrial genomes (Table 3). The other species were collected during several sampling campaigns (Curini-Galletti et al. 2012; Todaro et al. 2012, 2014, 2017, 2019). In brief, from freshwater samples, gastrotrichs were obtained by stirring the samples with a plastic pipette, and aliquots of the sediment–water mixture were decanted into 9 cm diameter plastic Petri dishes and analyzed under Wild-M8 stereo microscope. Individual gastrotrichs were picked with a glass micro-pipette and mounted on a slide in a drop of 1% MgCl_2_ solution to be analyzed under compound light microscope. For marine samples, one to two spoons of the fauna-enriched top layer of sandy sample were placed into a small vessel with a 7% MgCl_2_ added to cover the sand (as it is described in Todaro et al. 2019). The material is then swirled and allowed to sit for 5 min. After this, the supernatant was decanted into Petri dishes and analyzed under stereo microscope, and individual gastrotrichs were mounted on a slide for further analysis, as it was done for freshwater species.

**Table 3:** Specimens used in this study, with notes on classification, sampling locations, ecology, mtDNA GenBank accession codes, and references

Species	Classification	Sampling location	Reproduction/ecology	GenBank accession/references
*Aspidiophorus tentaculatus*	Chaetonotidae, Oiorpata, Chaetonotida	Carlotto, IT 41°13′44.56″N, 9°22′31.44″E	Parthenogenetic, marine	PX661610
*Chaetonotus* sp.	Chaetonotidae, Oiorpata, Chaetonotida	Pisa, IT 43°43′16.60″N, 10°23′45.75″E	Parthenogenetic, freshwater	PX661611
*Chaetonotus neptuni*	Chaetonotidae, Oiorpata, Chaetonotida	Asinara, IT 40° 59′41.28″N, 8° 12′50.76″E	Parthenogenetic, marine	PX661597
*Chaetonotus schultzei*	Chaetonotidae, Oiorpata, Chaetonotida	San Rossore, IT 43°43′12,33″N, 10°17′6,02″E	Parthenogenetic, freshwater	PX661598
*Chaetonotus apolemmus*	Chaetonotidae, Oiorpata, Chaetonotida	Asinara, IT 41°0′47.959″N, 8°14′56.306″E	Parthenogenetic, marine	PX661612
*Lepidodermella squamata*	Chaetonotidae, Oiorpata, Chaetonotida	Purchased from Carolina Biological Supply	Parthenogenetic, freshwater	Golombek et al. (2015)
*Litigonotus ghinii*	Chaetonotidae, Oiorpata, Chaetonotida	Pisa, IT 43°43′16.60″N, 10°23′45.75″E	Parthenogenetic, freshwater	Gammuto et al. (2024)
*Setopus* sp.	Dasydytidae, Oiorpata, Chaetonotida	Pisa, IT 43°43′16.60″N, 10°23′45.75″E	Parthenogenetic, freshwater	PX661599
*Xenotrichula intermedia 1*	Xenotrichulidae, Chaetonotida	Liguria, IT 44°02′57.66″N 9°58′53.51″E	Hermaphroditic, marine	PX661603
*Xenotrichula intermedia 2*	Xenotrichulidae, Chaetonotida	Milano Marittima, IT 44°16′41″N 12°20′53″E	Hermaphroditic, marine	PX661602
*Neodasys* sp.	Neodasyidae, Chaetonotida	SurfBeach, Panama, 7°25′51.6″N, 80°11′45.599″W	Hermaphroditic, marine	PX661604
*Anandrodasys agadasys*	Redudasyidae, Macrodasiyda	St John Island, USA 18°21′50″N; 64°43′47″W	Parthenogenetic, marine	PX661600
*Dolichodasys* sp.	Cephalodasyidae, Macrodasiyda	Sicily, IT 37°25′58″N; 13°14′29″E	Hermaphroditic, marine	PX661601
*Macrodasys meristocytalis*	Macrodasyidae, Macrodasiyda	Duncans, JM 18°29′13″N; 77°32′03″W	Hermaphroditic, marine	PX661605
*Megadasys* sp.	Planodasyidae, Macrodasiyda	Lanzarote, ES 28°55′08″N; 13°40′06″W	Hermaphroditic, marine	PX661606
*Paradasys* sp.	Cephalodasyidae, Macrodasiyda	Sardegna, IT 41°03′9″N; 8°56′16″E	Hermaphroditic, marine	PX661607
*Paraturbanella pallida*	Turbanellidae, Macrodasiyda	Sardegna, IT 41°16′43″N; 09°21′28″E	Hermaphroditic, marine	PX661608
*Turbanella ambronensis*	Turbanellidae, Macrodasiyda	Sicilia, IT 36°47′18″N; 14°29′34″E	Hermaphroditic, marine	PX661609
*Urodasys bifidostylis*	Macrodasyidae, Macrodasiyda	Sardegna, IT 41°03′9″N; 8°56′16″E	Hermaphroditic, marine	PX661613
*Urodasys mirabilis*	Macrodasyidae, Macrodasiyda	Willemstad, Curaçao 12°07′19″N; 68°58′09″W	Hermaphroditic, marine	PX661614
*Urodasys apuliensis*	Macrodasyidae, Macrodasiyda	Sardegna, IT 41°16′43″N; 09°21′28″E	Hermaphroditic, marine	PX661615
*Urodasys acanthostylis*	Macrodasyidae, Macrodasiyda	Lanzarote, ES 28°55′08″N; 13°40′06″W	Hermaphroditic, marine	PX661596
*Urodasys viviparus*	Macrodasyidae, Macrodasiyda	Abruzzo, IT 42°40′44″N; 14°01′05″E	Parthenogenetic, marine	PX661616

The morphological identification was conducted on living, relaxed specimens under a Nikon eclipse 90i or a Leitz Dialux 20 microscope equipped with differential interference contrast optics and fitted with a Nikon DS-Fi3 camera operated by a NIS-Elements D software (v 4.60.00). After taking high-resolution photographs for vouchers (Fig. 2), the specimens were retrieved from the slides, transferred to 0.5 mL centrifuge tubes filled with 96% ethanol, and stored at −20 °C for later DNA analysis.

### DNA Extraction and Amplification

Ethanol-preserved specimens were washed in clean absolute ethanol, individually transferred into sterile 0.5 mL tubes using a glass micro-pipette and left overnight at 25 °C in a cleaned ISCO micra 18 incubator to eliminate any residual ethanol through evaporation. Subsequently, 4 μL of phosphate-buffered saline solution was added to each sample. The samples were then processed for DNA extraction and WGA using the REPLI-g Single Cell Kit (QIAGEN) following the manufacturer's instructions (ie lysis and denaturation at room temperature at 65 °C for 10 min, amplification with incubation at 30 °C for 8 h, and inactivation of DNA Polymerase at 60 °C for 3 min). The resulting amplified DNA product was validated for the presence of gastrotrich DNA footprint through polymerase chain reaction (PCR) amplification of the 18S rDNA gene and Sanger sequencing. For each 40 µL PCR volume 2 µL of 1:100 diluted DNA template, 29.38 µL of water, 4 µL of reaction buffer, 4 µL of dNTPs in solution, 0.4 µL of paired primers, and 0.22 µL of Takara Taq-polymerasys was used. The primer combinations and thermal cycler program used for validation PCR are available in Supplementary Material S5. The PCR products were purified with the Monarch PCR & DNA Cleanup Kit (New England BioLabs Inc., Ipswich, MA, USA) and subsequently sent for Sanger sequencing to the Macrogen Europe Laboratory in Milan, Italy. Reads resulting from the Sanger sequencing were assembled manually using BioEdit (Hall 1999) into almost complete 18S rDNA sequences and examined with GenBank online Blast tool (https://blast.ncbi.nlm.nih.gov/Blast.cgi). The validated WGA DNA samples were then sent to Macrogen Europe (https://www.macrogen-europe.com/) and processed with a TrueSeq DNA PCR Free Library kit and de novo whole-genome sequencing at NovaSeq 6000 Illumina Platform to generate a total of 40 million reads (paired-ends 2×150 bp) for each sample.

### Mitochondrial Genome Assembly and Annotation

The obtained sequence data were analyzed through the Blobology pipeline followed by Gammuto et al. (2024). In brief, the quality of the reads was evaluated through the FASTQC software (Andrews 2010). The reads were then trimmed for quality and adapters whenever it was needed with TRIMMOMATIC 0.39 (Bolger et al. 2014) setting the minimum length to 140 bp and leaving the other parameters as default. The remaining paired reads were assembled through the SPAdes v.3.6.0 software (Bankevich et al. 2012) to obtain a preliminary assembly of the whole genomic content of the sample (ie gastrotrich nuclear genome, gastrotrich mitochondrial genome and eventually associated bacteria and ingested food). The assembled contigs matching to mitochondrial genes were identified through tBlastn analysis using sequences of protein-coding genes of *Lepidodermella squamata* (GenBank acc. KP965862) and *Litigonotus ghinii* (GeneBank acc. PP105008) mitochondrial genomes as queries. Where needed (ie in case, the whole mitochondrial genome was not assembled in a single circular contig), reads mapped to the so identified contigs were extracted from the original set and separately assembled using SPAdes in order to obtain the whole mitochondrial genome in a single contig. Ultimately, all mitogenomes were assembled as single contigs, except for four species in which two overlapping nodes were manually joined and confirmed to form circular genomes. Prediction and annotation of mitochondrial genes were performed using MITOS2 (Bernt et al. 2013) integrated in the Galaxy platform with the RefSeq63 Metazoan dataset and with the invertebrate mitochondrial genetic code. Annotated gene boundaries were checked and fixed manually through alignments, Blastn, Blastp, and NCBI ORF finder when necessary. Additionally, tRNA genes were predicted using *tRNAscan-SE* (Lowe and Chan 2016) with the invertebrate mitochondrial code, and results were cross-validated through manual inspection and comparison with MITOS2 predictions.

To verify the accuracy of the assemblies for *U. apuliensis* and *U. mirabilis*, as well as the annotation of their mtDNA (for which a potential gene duplication events were observed), their WGA templates were re-sequenced using Oxford Nanopore technology, and the results were compared. A second specimen of *X. intermedia* from a different population was investigated using the Oxford nanopore technology to look for possible differences due to technology and/or population genetics. For Oxford nanopore sequencing, first the amplified genomic DNA was cleaned with AMPure XP beads and then processed for endonuclease digestion using a T7 Endonuclease digestion kit (adapted from Lee et al. 2023). Next, the product was processed for native barcoding and adapter ligation using Native Barcoding Kit 24V14. The reads were assembled with Fly v.2.9.5 following the protocol of Lee et al. (2023). Gene duplication detected in *U. apuliensis* and *U. mirabilis* during the reads assembly was later confirmed by comparison of crystal structure (obtained with Alphafold3, Abramson et al. 2024) and specific core regions conserved across all sequences. In subsequent phylogenetic analyses, only the gene copy with the best alignment fit was used for these two species presenting duplications.

To confirm completeness and circularity of mtDNA in specimens where the start and end of the assembled sequences did not overlap (*n* = 7 out of 21), we inferred completeness based on gene content and genome structure. As a proof of concept, we designed PCR primers targeting the two free extremities of two representative linear assemblies. Successful amplification across the predicted junction confirmed their circular topology (see details in Supplementary Material S6).

### ATP Gene Search in Macrodasyidan Datasets

To investigate the presence/absence of *atp* genes in the order Macrodasyida, we employed both a heuristic approach (Blast; Camacho et al. 2009) and an HMM-based method (Exonerate; Slater and Birney 2005). In both cases, *atp* sequences from Chaetonotida were used as queries against Macrodasyida assemblies, and a completeness matrix (presence/absence) was generated (Supplementary Material S4). To test whether *atp* has translocated from the mitochondrion to the nucleus, we analyzed the scaffolds containing *atp* using MITOS2 (Bernt et al. 2013). As an additional validation, the putative *atp* sequences were compared against the nonredundant protein database (nr) using Blastx, confirming their identity as *atp*. Protein integrity analysis GetOrf (EMBOSS, Rice et al. 2000) was used to check the length of functional *atp* sequences. TargetP-2.0 (Emanuelsson et al. 2000) and MitoProt (Claros and Vincens 1996) web-based tools were used to assess the presence of mitochondrial targeting signals in *atp* sequences.

### Phylogenetic Analyses

Mitochondrial protein-coding genes were used for the phylogenetic analysis. The mitochondrial amino acid sequences obtained from MITOS2 annotation output were aligned separately for each gene with MEGA X, using the integrated Muscle algorithm (Kumar et al. 2018). Next, all the alignments were concatenated into a final single matrix using MEGA X, resulting in 4,150 amino acid sites. Additionally, two free-living plathyhelminth species *Nematoplana* sp. (LC760198) and *Macrostomum lignano* (MF078637) were added to this analysis as an out-group. Phylogenetic trees were built using ML and BI approaches. ML analyses were performed in IQ-TREE v.1.6.10 (Nguyen et al. 2015) with the following settings and considerations: (i) we used the best-fit partition models according to BIC (Bayesian information criterion) for 13 amino acid dataset identified by IQ tree (see Supplementary Material S7), (ii) edge-linked partition option, (iii) 1,000 ultrafast bootstrap pseudo-replicates with the SH-aLRT support activated to ensure additional confirmation for Ultrafast bootsrap values (ie to consider clade confident with the values SH-aLRT ≥ 80% and UFbootstrap ≥ 95%), and (iv) the rest of the parameters were left as default. For the Bayesian analyses, the amino acid dataset was run in the program MrBayes v.3.2.7 (Ronquist et al. 2012) with 200,000 generations, with gamma distribution across invariable sites and fixed mtrev amino acid substitution model, with a sampling frequency of trees and parameters at 100, and with a relative burn-in fraction of 25%. Convergence of the MCMC analyses was confirmed with the in-built diagnostics of the program with the average standard deviation of split frequencies was 0.006792, the potential scale reduction factor converged to 1.00 for all parameters, the effective sample sizes (ESSs) of all parameters were >200 (ie min. ESS = 5.44E + 08, av. ESS = 5.54E + 08). The ML and BI trees were computed as unrooted and then were rooted in FigTree v.1.4.3 (http://tree.bio.ed.ac.uk/software/figtree/), with the flatworm species used as out-group. Since both methods generated the same topology, ML tree is presented combining both Bootstrap (BB) and PP support values obtained from both methods. The final tree was edited for better visualization using CorelDraw X7 (Corel Corporation, Ottawa, Canada). As the number of protein-coding genes varied among the analyzed species, we ran an additional phylogenetic analysis involving solely the genes present in all the investigated species, looking for possible effects of gene sampling (Supplementary Material S1).

### Mitochondrial Genome Structural Analysis

Mitochondrial genome structure and specificity were analyzed by using several tools. The length of the mitochondrial genomes, number of the protein-coding genes, their position, order (gene synteny), and transcriptional direction were observed from annotation reports generated by MITOS2 integrated in Galaxy platform (Al Arab et al. 2017; Donath et al 2019; The Galaxy Community 2024) and compared through species. Mitochondrial genome base count and GC content were calculated using BIC online calculator (https://www.biologicscorp.com/tools/GCContent/). Repeated elements were identified with Tandem Repeats Finder (Benson 1999) online tool (https://tandem.bu.edu/trf/home). Codon usage analysis was done using MEGA X software package (Kumar et al. 2018). The mitochondrial genome maps were created using circularMT toolkit (Goodman and Carr 2024). To examine the relationship between mitochondrial genome features and phylogenetic groupings, we conducted a series of statistical tests in PAST version 5.2.1 (Hammer et al. 2001). Specifically, we assessed intergroup differences in GC content and repeat number and evaluated correlations among mitochondrial traits. A Student's *t*-test was performed to compare GC content between the two orders. To assess differences in tandem repeat content, we employed the nonparametric Mann–Whitney *U* test, as repeat counts did not conform to normality assumptions. We further explored the potential association between genome architecture and sequence composition by applying Spearman's rank correlation tests between (i) GC content and tandem repeat abundance and (ii) GC content and transcriptional directionality (quantified as the proportion of genes encoded on the same strand).

## Supplementary Material

evag001_Supplementary_Data

## Data Availability

Results of all analyses are available in the article and in its online Supplementary material. Mitochondrial genome sequences are available in the GenBank Nucleotide Database (https://www.ncbi.nlm.nih.gov/nucleotide/), with accession numbers PX661596 to PX661616. Additionally, the alignments used to generate phylogenetic trees, as well as the mitochondrial genome sequences with their annotations, have been uploaded to Figshare (https://figshare.com/) and are available at the following DOIs: 10.6084/m9.figshare.29414720 and 10.6084/m9.figshare.30788585.
