# Supplementary material for: From Rigid Order to Radical Variation: Mitogenome Evolution in the Main Lineages of a Lesser-Known Animal Phylum (Gastrotricha)
Source: Genome Biol Evol. 2026 Jan 10;18(2):evag001. doi: 10.1093/gbe/evag001 (PMC12862867; doi:10.1093/gbe/evag001)
Supplement: evag001_Supplementary_Data [file evag001_supplementary_data.pdf]

Supplementary Material

From rigid order to radical variation: mitogenome evolution in the main lineages of a lesser-known animal phylum (Gastrotricha)

Anush Kosakyan[1,2*] † , Leandro Gammuto[3] † , Agata Cesaretti[1], Francesco Saponi[1,2,4], Valentina Serra[5], Giulio Petroni[5,6,7], Jan-Niklas Macher[8,9], Oscar Wallnoefer[10], Federico Plazzi[10], M. Antonio Todaro[1,2]

[1]Department of Life Sciences, University of Modena and Reggio Emilia, Modena, Italy
[2]National Biodiversity Future Center (NBFC), Palermo, Italy
[3]Department of Biology and Biotechnology "Lazzaro Spallazani", University of Pavia, Pavia, Italy
[4]Department of Earth and Marine Sciences, University of Palermo, Palermo, Italy
[5]Dipartimento of Biology, University of Pisa, Pisa, Italy
[6] Interdepartmental Center for Electron Microscopy (CIME), University of Pisa, Pisa, Italy
[7] Center for Instrument Sharing of the University of Pisa (CISUP), University of Pisa, Pisa, Italy
[8]Naturalis Biodiversity Center, Leiden, Netherlands
[9]Department of Environmental Biology, Institute of Environmental Sciences (CML), Leiden University, Leiden, The Netherlands
[10]Department of Biological, Geological and Environmental Sciences, University of Bologna, Bologna, Italy

Suppl. Mat. 1. Comparing phylogenetic results based on 13 mt PCGs and 11 mt PCGs (when excluding *atp6* and *atp8* genes from concatenated dataset).

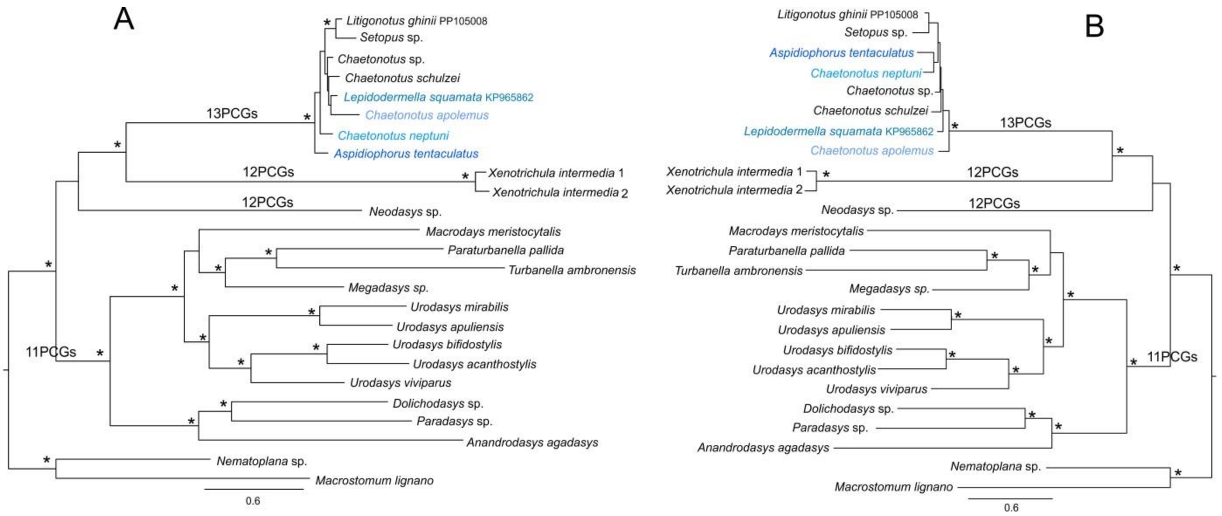

IQ ML tree showing phylogenetic relationships of 22 gastrotrich species and two flatworm species (used as outgroup) based on 13 mitochondrial protein coding gene (*cox1-3*, *cob*, *nad1-6*, *nad4L, atp6, atp8*) concatenated alignment in **A** and IQ tree showing phylogenetic relationships of 22 gastrotrich species and two flatworm species (used as outgroup) based on 11 mitochondrial

protein coding gene (*cox1-3*, *cob*, *nad1-6*, *nad4L*) concatenated alignment in **B**. The terminals with altered positions are indicated in blue. Nodes are indicated with * when SHaLRT support is > 85% and when Ultrafast Bootstrap support is > 95%. The scale bar indicates the number of substitutions per site. The information on number of protein coding genes (PCGs) of mtDNA for representatives of each lineage is presented on the branches.

Suppl. Mat. 2. The comparision of mtDNA base calculation across studied species.

**Species**	**mtDNA base calculation**	**GC %**
Aspidiophorus tentaculatus	Full Length(14560bp) | A(22% 3221) | T(40% 5561) | G(22% 3304) | C(16% 2474)	38
Chaetonotus sp.	Full Length(14488bp) | A(21% 3108) | T(39% 5480) | G(23% 3340) | C(17% 2560)	40
Chaetonotus neptuni	Full Length(14585bp) | A(23% 3407) | T(40% 5646) | G(22% 3239) | C(15% 2293)	37
Chaetonotus schultzei	Full Length(14503bp) | A(20% 3044) | T(40% 5535) | G(23% 3398) | C(17% 2526)	40
Chaetonotus apolemmus	Full Length(14599bp) | A(21% 3118) | T(39% 5431) | G(23% 3481) | C(17% 2569)	40
Lepidodermella squamata	Full Length(14558bp) | A(21% 3113) | T(39% 5454) | G(22% 3338) | C(18% 2653)	40
Litigonotus ghinii	Full Length(14384bp) | A(20% 2945) | T(38% 5271) | G(24% 3529) | C(18% 2639)	42
Setopus sp.	Full Length(14495bp) | A(20% 2952) | T(38% 5298) | G(24% 3604) | C(18% 2641)	42
*Xenotrichula intermedia*1	Full Length(15103bp) | A(26% 4021) | T(38% 5586) | G(19% 2880) | C(17% 2616)	31
*Xenotrichula intermedia*2	Full Length(14919bp) | A(27% 4164) | T(42% 5902) | G(17% 2672) | C(14% 2181)	31
Neodasys sp.	Full Length(14156bp) | A(22% 3155) | T(38% 5303) | G(22% 3124) | C(18% 2574)	40
Anandrodasys agadasys	Full Length(16272bp) | A(45% 7326) | T(26% 4071) | G(10% 1682) | C(19% 3193)	29
Dolichodasys sp.	Full Length(15893bp) | A(38% 6066) | T(36% 5564) | G(12% 1989) | C(14% 2274)	26
Macrodasys meristocytalis	Full Length(14402bp) | A(40% 5767) | T(31% 4346) | G(10% 1510) | C(19% 2779)	29
Megadasys sp.	Full Length(14487bp) | A(35% 5160) | T(41% 5725) | G(11% 1631) | C(13% 1971)	24
Paradasys sp.	Full Length(12838bp) | A(37% 4823) | T(38% 4683) | G(13% 1719) | C(12% 1613)	25
Paraturbanella pallida	Full Length(14981bp) | A(38% 5796) | T(42% 6149) | G(10% 1505) | C(10% 1531)	20
Turbanella	Full Length(14297bp) | A(21% 3104) | T(33% 4522) | G(24% 3515) |	**46**

ambronensis	C(22% 3156)	
Urodasys bifidostylis	Full Length(15624bp) | A(39% 6195) | T(40% 6031) | G(8% 1334) | C(13% 2064)	21
Urodasys mirabilis	Full Length(19009bp) | A(38% 7224) | T(31% 5685) | G(10% 1956) | C(21% 4144)	31
Urodasys apuliensis	Full Length(18723bp) | A(36% 6867) | T(42% 7599) | G(12% 2323) | C(10% 1934)	22
Urodasys acanthostylis	Full Length(15504bp) | A(39% 6070) | T(39% 5939) | G(13% 2084) | C(9% 1411)	22
Urodasys viviparus	Full Length(13340bp) | A(41% 5504) | T(39% 5012) | G(9% 1303) | C(11% 1521)	20

Suppl. Mat. 3. Detailed information on tandem repeats identified in studied mtDNA by TandemRepeatsFinder. The table contains the following information: Indices of the repeat relative to the region of the sequence, Period size of the repeat, Number of copies aligned with the consensus pattern, Size of consensus pattern (may differ slightly from the period size), Percent of matches between adjacent copies overall, Percent of indels between adjacent copies overall, Alignment score, Percent composition for each of the four nucleotides, Entropy measure based on percent composition.

Aspidiophorus tentaculatus

Indices	Period Size	Copy Number	Consensus Size	Percent Matches	Percent Indels	Score	A	C	G	T	Entropy (0-2)
13842--13869	14	2.0	14	100	0	56	28	7	35	28	1.84

Chaetonotus neptuni

Indices	Period Size	Copy Number	Consensus Size	Percent Matches	Percent Indels	Score	A	C	G	T	Entropy (0-2)
8052--8105	27	2.0	27	100	0	108	29	29	22	18	1.97

Chaetonotus schultzei

Indices	Period Size	Copy Number	Consensus Size	Percent Matches	Percent Indels	Score	A	C	G	T	Entropy (0-2)
66--98	9	3.7	9	95	0	57	60	0	6	33	1.21

Lepidodermella squamata

Indices	Period Size	Copy Number	Consensus Size	Percent Matches	Percent Indels	Score	A	C	G	T	Entropy (0-2)
52--94	2	21.5	2	100	0	86	51	0	48	0	1.00

Anandrodasys agadasys

Indices	Period Size	Copy Number	Consensus Size	Percent Matches	Percent Indels	Score	A	C	G	T	Entropy (0-2)
116--140	13	1.9	13	100	0	50	8	88	4	0	0.64
5608--5656	26	1.9	26	87	4	73	71	12	10	6	1.30

Macrodasys meristocytalis

Indices	Period Size	Copy Number	Consensus Size	Percent Matches	Percent Indels	Score	A	C	G	T	Entropy (0-2)
2088--2126	18	2.2	18	95	0	69	64	17	0	17	1.30
2690--2897	43	4.8	43	98	0	398	41	1	7	49	1.41
2807--2967	69	2.3	70	87	5	229	42	1	7	48	1.42
2868--2967	25	4.2	25	70	17	104	43	1	9	47	1.41
2982--3035	17	3.2	17	100	0	108	29	0	11	59	1.32
2892--3001	45	2.4	46	95	4	197	40	1	7	50	1.41
3017--3095	25	3.2	25	72	3	88	41	1	7	49	1.39
2982--3047	34	1.9	34	87	0	96	34	0	10	54	1.35
2936--	83	2.0	80	87	6	234	39	1	8	51	1.39

3096											

Megadasys sp.

Indices	Period Size	Copy Number	Consensus Size	Percent Matches	Percent Indels	Score	A	C	G	T	Entropy (0-2)
918--1012	49	1.9	49	100	0	190	41	4	0	54	1.20
944--1014	15	4.5	15	68	13	52	39	2	0	57	1.13
5935--5964	16	1.9	16	93	6	53	23	0	0	76	0.78
8006--8053	21	2.3	21	89	7	71	77	4	0	18	0.93
12979--13020	21	1.9	23	90	9	70	45	0	2	52	1.13

Paradasys sp.

Indices	Period Size	Copy Number	Consensus Size	Percent Matches	Percent Indels	Score	A	C	G	T	Entropy (0-2)
1190--1226	18	2.0	19	94	5	67	40	21	21	16	1.91
5885--5951	30	2.2	29	80	17	75	34	8	0	56	1.31

Paraturbanella pallida

Indices	Period Size	Copy Number	Consensus Size	Percent Matches	Percent Indels	Score	A	C	G	T	Entropy (0-2)
2485--2726	121	2.0	121	100	0	484	33	7	15	42	1.75
2838--2890	24	2.2	24	93	0	88	39	0	1	58	1.09
2831--	15	3.8	15	69	28	51	41	0	0	58	0.98

2881											
5350--5382	15	2.2	15	100	0	66	39	0	0	60	0.97
6605--6642	20	1.9	20	84	10	51	21	5	2	71	1.19
10383--10435	22	2.3	23	80	3	63	52	1	0	45	1.11
13213--13241	15	1.9	15	100	0	58	68	6	0	24	1.13

Turbanella ambronensis

Indices	Period Size	Copy Number	Consensus Size	Percent Matches	Percent Indels	Score	A	C	G	T	Entropy (0-2)
13653--14297	239	2.7	239	89	3	1035	27	26	21	24	1.99

Urodasys bifidostylis

Indices	Period Size	Copy Number	Consensus Size	Percent Matches	Percent Indels	Score	A	C	G	T	Entropy (0-2)
1--1278	237	5.8	215	81	10	1350	39	11	8	40	1.72
160--1276	444	2.5	445	88	3	1634	38	11	8	40	1.72
621--1035	207	2.0	207	89	1	636	37	13	8	40	1.75
828--1409	237	2.5	237	89	3	861	40	11	7	41	1.68
6869--6895	14	1.9	14	100	0	54	74	3	0	22	0.98
8132--8175	14	3.1	14	80	0	61	79	11	2	6	1.01
1469	240	3.9	239	90	3	147	3	1	7	4	1.71

Indices	Period Size	Copy Number	Consensus Size	Percent Matches	Percent Indels	Score	A	C	G	T	Entropy (0-2)
4--15624						8	8	1		1	
14678--15624	480	2.0	479	98	0	1815	38	11	7	41	1.70

Urodasys mirabilis

Indices	Period Size	Copy Number	Consensus Size	Percent Matches	Percent Indels	Score	A	C	G	T	Entropy (0-2)
11463--11500	14	2.5	15	79	12	51	71	21	2	5	1.19
13232--13288	24	2.5	22	78	16	53	70	5	3	21	1.22

Urodasys apuliensis

Indices	Period Size	Copy Number	Consensus Size	Percent Matches	Percent Indels	Score	A	C	G	T	Entropy (0-2)
1--2590	164	16.0	161	88	3	3552	39	6	10	43	1.65
1--2956	485	6.2	485	87	5	3981	39	6	10	43	1.65
2392--2957	142	4.0	143	82	6	688	39	7	10	43	1.66
2392--2957	283	2.0	281	83	7	709	39	7	10	43	1.66

Urodasys acanthostylis

Indices	Period Size	Copy Number	Consensus Size	Percent Matches	Percent Indels	Score	A	C	G	T	Entropy (0-2)
10316--10348	16	2.0	17	88	5	50	42	6	9	42	1.61
10704--1073	13	2.1	14	93	6	51	51	0	0	48	1.00

2											

Urodasys viviparus

Indices	Period Size	Copy Number	Consensus Size	Percent Matches	Percent Indels	Score	A	C	G	T	Entropy (0-2)
423--474	25	2.1	25	100	0	104	69	0	7	23	1.14
434--486	25	2.2	24	83	6	63	73	0	9	16	1.08
1841--1875	11	3.1	11	91	4	52	37	0	0	62	0.95
1840--1899	12	4.6	12	75	17	57	33	0	1	65	1.03
1847--1911	29	2.3	28	76	7	69	44	0	1	53	1.09
2060--2099	19	2.2	18	86	13	55	32	2	5	60	1.32
4792--4818	7	3.9	7	100	0	54	44	0	0	55	0.99
6228--6257	9	3.3	9	95	0	51	13	0	0	86	0.57

Suppl. Mat. 4. *atp6* presence/absence matrix in macrodasyidan draft assemblies (nuclear contigs) using Blast and Exonerate search with chaetonotodan atp6 sequences as queries. Dark blue color suggests stronger hit (highest homology) of chaetonotidan atp6 quiery sequences in studied macrodasyidan nuclear contigs. White color indicates no hit. *atp6* was found in two different *T. ambronensis* nuclear contigs reported here as *T. ambronensis* 1 and 2.

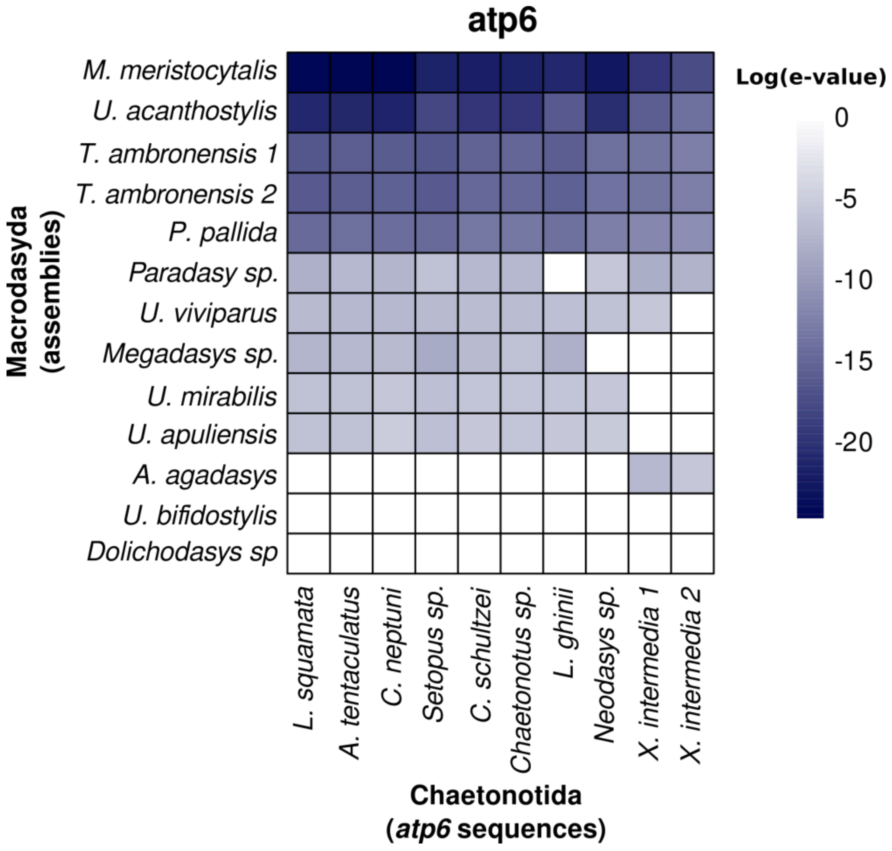

Suppl. Mat. 5 Details on primers and PCR programs used for amplification and sequencing of the 18S rDNA gene in the validation step.

**Primer**	**Direction**	**Sequence 5' to 3'**	**Usage**	**Reference**
S30	forward	GCTTGTCTCAAAGATTAAGCC	PCR	Norén and Jondelius 1999
1806R	reverse	CCTTGTTACGACTTTTACTTCCTC	PCR	Norén and Jondelius 1999
18S R536	reverse	CTGGAATTACCGCGGCTG	sequencing	Rosati *et al.* 2004
18S R1052	reverse	AACTAAGAACGGCCATGCA	sequencing	Rosati *et al.* 2004
18S F783	forward	GACGATCAGATACCGTC	sequencing	Rosati *et al.* 2004

Details of PCR protocol: 3 min at 95 °C, 35 × (30 s at 94 °C, 30 s at 50 °C, 2 min at 72 °C), 7 min at 72 °C

Suppl. Mat. 6 Details of primers and schematic representation of the PCR design that was used to close the mitochondrial DNA of *Urodasys mirabilis* and Anandrodasys agadasys. For each species, we designed two pairs of primers that allowed three different products: one pair of primers amplified a known region in the extreme "A", while another pair amplified the extreme "B". Additionally, the forward primer from extreme A also worked with the reverse primer from extreme B, ensuring the functionality of each primer. As a control, we included *Chaetonotus schultzei*, a species previously confirmed to have circular mtDNA through bioinformatic analysis.

**Primer Code**	**Primer Sequence (5'-3')**
Umir_Fwd_A	TATAGATGCCAACCCCCACC
Umir_Rvs_B	AGAAGTCACCACAAAGCCGA
Umir_Rvs_A	GGTGGAGTGCTGATAGACGA
Umir_Fwd_B	CCGAAATTACAGGAGAGGGTT
Csch_Fwd_A	ATGCCCCGACGATATAGGGA
Csch_Rvs_B	GGACAACAACACCTAGCCCA
Csch_Rvs_A	AAAGGCAGCACGTCTTCTCA
Csch_Fwd_B	AAATGGCTGAAGTTAGGCGGA
Aaga_Fwd_A	TCTCTAACCGCTGTGCAAGAA
Aaga_Rvs_B	ACCCTAGCCAATTAGTGGTGAT
Aaga_Rvs_A	GGGTTCCCTATGGGCCTTGT
Aaga_Fwd_B	AGCTACCGAGAACGTAGGGT

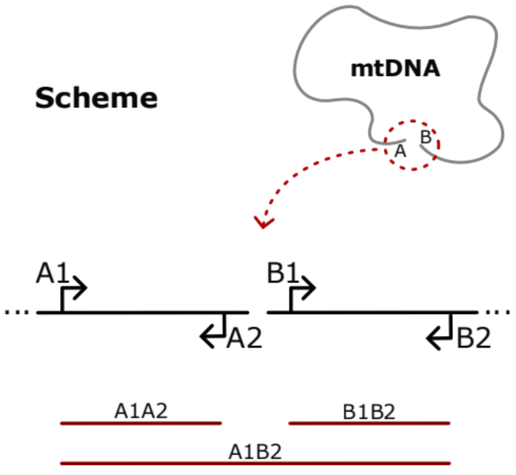

Amplified region

	**A1A2**	**A1B2**	**B1B2**
U. mirabilis	269	499	193
C. schultzei	194	694	207
A. agadasys	208	736	215

Suppl. Mat. 7. Partition models used for phylogenetic analyses

The concatenated amino acid alignment was partitioned as following

charset part1=1-550; for *cox1*
charset part2=551-817; for *cox2*
charset part3=818-1100; for *cox3*
charset part4=1101-1497; for *cob*
charset part5=1498-1825; for *nad1*
charset part6=1826-2272; for *nad2*
charset part7=2273-2413; for *nad3*
charset part8=2414-2937; for *nad4*
charset part9=2938-3062; for *nad4l*
charset part10=3063-3667; for *nad5*
charset part11=3668-3856; for *nad6*
charset part12=3857-4095; for *atp6*
charset part13=4096-4150; for *atp8*

ModelFinder integrated in IQ platform suggested the following model combination

Best-fit model according to BIC:
mtZOA+G4:part1,mtZOA+F+I+G4:part2,mtZOA+I+G4:part3,mtZOA+I+G4:part4,mtART+I+G4:part5,mtInv+I+G4:part6,mtART+I+G4:part7,mtInv+I+G4:part8,mtInv+G4:part9,mtInv+I+G4:part10,mtInv+G4:part11,mtZOA+G4:part12,mtInv+G4:part13

List of best-fit models per partition:

ID	Model	LogL	AIC	w-AIC	AICc	w-AICc	BIC	w-BIC
1	mtZOA+G4	-11797.7492	23691.4984	+ 0.0000	23700.8877	+ 0.0000	23898.3745	+ 0.0000
2	mtZOA+F+I+G4	-7403.0891	14942.1781	+ 0.0000	14989.5721	+ 0.0000	15186.1111	+ 0.0000
3	mtZOA+I+G4	-8049.4672	16196.9343	+ 0.0000	16217.9644	+ 0.0000	16375.5612	+ 0.0000
4	mtZOA+I+G4	-11402.7812	22903.5623	+ 0.0000	22917.6834	+ 0.0000	23098.7752	+ 0.0000
5	mtART+I+G4	-9298.9242	18695.8484	+ 0.0000	18713.4743	+ 0.0000	18881.7061	+ 0.0000
6	mtInv+I+G4	-12095.4359	24288.8717	+ 0.0000	24301.2143	+ 0.0000	24489.8971	+ 0.0000
7	mtART+I+G4	-4055.3547	8208.7095	+ 0.0000	8262.5556	+ 0.0000	8353.1987	+ 0.0000
8	mtInv+I+G4	-15363.7711	30825.5422	+ 0.0000	30835.8798	+ 0.0000	31034.3553	+ 0.0000
9	mtInv+G4	-3642.9138	7381.8275	+ 0.0000	7443.7223	+ 0.0000	7517.5866	+ 0.0000
10	mtInv+I+G4	-17579.1137	35256.2274	+ 0.0000	35265.0562	+ 0.0000	35472.0836	+ 0.0000
11	mtInv+G4	-6212.1887	12520.3774	+ 0.0000	12553.9774	+ 0.0000	12675.9813	+ 0.0000
12	mtZOA+G4	-3462.9865	6973.9730	+ 0.0000	6979.5804	+ 0.0000	7057.4081	+ 0.0000
13	mtInv+G4	-373.8154	779.6308	+ 0.0000	793.9466	+ 0.0000	811.7482	+ 0.0000

AIC, w-AIC: Akaike information criterion scores and weights.
AICc, w-AICc: Corrected AIC scores and weights.
BIC, w-BIC: Bayesian information criterion scores and weights.

Plus signs denote the 95% confidence sets.
Minus signs denote significant exclusion.
